# Tau Oligomers and Fibrils Exhibit Differential Patterns of Seeding and Association With RNA Binding Proteins

**DOI:** 10.3389/fneur.2020.579434

**Published:** 2020-09-30

**Authors:** Lulu Jiang, Jian Zhao, Ji-Xin Cheng, Benjamin Wolozin

**Affiliations:** ^1^Department of Pharmacology and Experimental Therapeutics, Boston University School of Medicine, Boston, MA, United States; ^2^Boston University Photonics Center, Boston, MA, United States; ^3^Center for Neurophotonics, Boston University School of Medicine, Boston, MA, United States; ^4^Department of Neurology, Boston University School of Medicine, Boston, MA, United States; ^5^Center for Systems Neuroscience, Boston University School of Medicine, Boston, MA, United States

**Keywords:** tau oligomer, neurofibrillary tangle (NFT), image proceeding, RNA translation, stress, stress granules, TIA1, PABP

## Abstract

Tau aggregates are pleiotropic and exhibit differences in conformation, structure, and size. These aggregates develop endogenously but are also propagated among neurons in disease. We explored the actions of two distinct types of tau aggregates, tau oligomers (oTau) and tau fibrils (fTau), using a seeding assay in primary neuron cultures expressing human 4R0N tau. We find that oTau and fTau elicit distinct patterns of tau inclusions in the neurons and distinct molecular interactions. The exogenously applied oTau and fTau both clear rapidly from the neurons, but both also seed intracellular inclusions composed of endogenously produced tau. The two types of seeds elicit differential dose–response relationships for seed uptake and the number of resulting intracellular inclusions. Immunocytochemical studies show that co-localization with RNA binding proteins associated with stress granules is much greater for seeds composed of oTau than fTau. Conversely, co-localization with p62/SQSTM1 and thioflavine S is much greater for fTau than oTau. These results suggest that oTau seeds inclusions that modulate the translational stress response and are physiologically active, whereas fTau seeds inclusions that are fibrillar and shunted to the autolysosomal cascade.

## Introduction

The microtubule association protein tau is one of the principal components of pathology in Alzheimer's disease and related disorders (ADRD) (1). Tau exists primarily in the axon under basal conditions, with smaller amounts in the dendritic arbor. However, stress induces phosphorylation of tau and localization to the somato-dendritic compartment (2, 3). As the stress persists, the tau begins to accumulate as oligomers and then fibrils, ultimately forming neurofibrillary tangles, which are a pathological hallmark of Alzheimer's disease and other tauopathies.

Our understanding of the patterns and properties of tau aggregates has evolved steadily. *In vitro* studies showed that tau exhibits an intrinsic affinity for RNA, an anionic agent, and a tendency to fibrillize in the presence of anionic agents, including RNA, heparin sulfate, dextran sulfate, and arachidonic acid (4–6). Aggregation of purified tau with anionic agents leads to an ordered assembly of tau into oligomers and then fibrils, with formation of oligomers thought to be rate limiting (7). However, the physiology of tau is quite different because stress induces phosphorylation of tau at proline directed serines and threonines, which accelerates oligomerization and subsequent fibrillization, and also leads to the somatodendritic accumulation of tau (8, 9).

Studies increasingly reveal remarkable differences in the properties of tau aggregates. The evidence showing that tau conformations differ among diseases is perhaps most evident in the multiple Cryo-EM studies that have been recently published, showing the structural differences among fibrils isolated from brains of subjects with Alzheimer's disease, Pick's disease, corticobasal disease, and chronic traumatic encephalopathy (10–14). Although tau fibrils are the pathological hallmarks of disease, some studies suggest that they are end-stage species that might not be the actual tau aggregate responsible for toxicity and neurodegeneration. Studies in transgenic mouse models of tauopathy demonstrate that neurofibrillary tangles do not induce degeneration on their own, and that neurons lacking neurofibrillary pathology exhibit more functional deficits than neurons containing neurofibrillary pathology (15–17).

Seeding and propagation studies provide a powerful means to compare the biological actions of different conformations of tau aggregates. Studies of tau propagation demonstrate the presence of strains of tau aggregates that differ in their pattern of propagation (18). Direct comparison of tau oligomers and fibrils using an *in vivo* propagation assay demonstrates that both species propagate robustly but that oligomeric tau (oTau) elicits much more neurodegeneration (19). This study also showed a striking association between tau oligomers and RNA binding protein (RBP) pathology (19).

Multiple studies demonstrate associations between tau and elements of RNA metabolism, including RBPs and ribosomal proteins (20–27). Tau binds to ribosomal proteins (23, 24, 28). Tau plays a role in the nucleolus, possibly regulating ribosome biogenesis during stress (29). Tau regulates the formation of stress granules (SGs), which are an important element of the translational stress response, and reducing TIA1, a core SG nucleating RBP, delays progression in a mouse model of tauopathy (20, 21). The link between tau and stress is particularly important because stress induces hyperphosphorylation, oligomerization, and somatodendritic translation of tau, and stress is an inherent element of the disease process. In contrast, tau fibrillization evolves more slowly and is less tightly linked to physiological responses to stress. These observations support a hypothesis that species such as tau oligomers function as part of an integrated stress response and affect neurons in a manner that differs from tau fibrils.

The current study directly compares the actions of oTau and fibrillar tau (fTau) using a seeding assay in primary neuron cultures expressing human 4R0N tau (tau containing 4 repeats in the microtubule binding domain and 0 repeats in the amino-terminal domain). We find oTau and fTau elicit distinct patterns of tau inclusions in the neurons even though the exogenously applied oTau and fTau clear rapidly from the neurons. We also observe that oTau inclusions co-localize strongly with RBPs but exhibit little thioflavine S reactivity, whereas fTau inclusions co-localize strongly with thioflavine S reactivity but weakly with RBPs. These results point to a distinct biological activity of oTau.

## Materials and Methods

### Animals

Use of all animals was approved by the Boston University Institutional and Animal Care and Use Committee. All animals were housed in an IACUC-approved vivarium at Boston University School of Medicine. Breeders of P301S/PS19 mice were obtained from the Jackson Lab (stock no. 008169). Generation F2 mice were bred in-house and aged to 9 months for experiment. Timed pregnant C57BL/6 were purchased from Charles River Laboratories and delivered at E-14, and then the postnatal P0 pups were used for primary hippocampal cultures.

### S1p and P3 Fractions Extraction From PS19 Brain Tissue

The frozen hippocampus and cortex tissues (100–250 mg) were weighed and placed in a Beckman polycarbonate thick-wall centrifuge tube (cat no. 362305). A 10 × volume of homogenization buffer was then added to homogenize brain tissue with TBS buffer (50 mM Tris, pH 8.0, 274 mM NaCl, 5 mM KCl) supplemented with protease and phosphatase inhibitor cocktails (Roche, cat no. 05892791001 and cat no. 04906837001), as described previously (20). The homogenate was then ultracentrifuged at 28,000 rpm for 20 min at 4°C. After that, the supernatant was aliquoted to new microfuge tubes as S1 fraction (TBS-soluble). Then the pellet (P1 fraction) was homogenized with buffer B (10 mM Tris, pH 7.4, 800 mM NaCl, 10% sucrose, 1 mM EGTA, 1 mM PMSF), which was ~5 × volume of wet weight of the original tissue. The P1 homogenate was then ultracentrifuged at 22,000 rpm for 20 min at 4 °C. Next, the supernatant (S2 fraction) was aliquoted to a new Beckman polycarbonate thick-wall tube and incubated with 1% Sarkosyl rotating in the bench top thermomixer at 37°C for 1 h. After the incubation, the fraction mix was ultracentrifuged at 55,000 rpm for 1 h at 4°C. Then the sarkosyl-insoluble pellet (P3 fraction) was resuspended with 50 μl TE buffer (10 mM Tris, 1 mM EDTA, pH 8.0). For the extraction of S1p fraction, the supernatant (S1) fractions were ultracentrifuged a second time at 55,000 rpm at 4°C for 40 min. The TBS-extractable pellet (S1p) fractions were then resuspended in 4 × volume of TE buffer relative to the starting weight of the tissue homogenate.

The molecular weight of tau in these two fractions (S1p and P3) was documented by native polyacrylamide gel electrophoresis as described previously (19). Briefly, the concentration of total tau was measured by immunoblot using 3–12% reducing SDS-PAGE gel by comparison with gradient concentrations of recombinant tau ladders, using the tau-5 antibody (detecting total tau) by immunoblot. All the fractions were then normalized and divided into fractions of 20 μg/ml tau for storage and future use.

### Primary Hippocampal Culture With P0 Pups

The sterilized 12-mm coverslips were placed into each well of a 24-well-plate and then coated with 1 mg/ml poly-D-lysine for 1 h at room temperature in the culture hood. Then the plates were washed three times with sterile biology-grade water and dried in hood overnight. For the dissection of hippocampus from P0 pups, the pups were anesthetized via hypothermia by wrapping in gauze and placing in aluminum foil pouch on ice. After the brain was isolated from the skull, the meninges need to be completely removed from the brain tissue and unfurl the hippocampus. Then all the hippocampi were transferred into 15-ml conical tubes with 5 ml 0.25% Trypsin–EDTA supplemented with 150 μl DNase. The brain tissue was incubated in a 37 °C water bath for 15 min before being resuspended and triturated in 2 ml plating medium (MEM Gibco no. 11090, 2.5% FBS, 1 × penicillin/streptomycin, l-glutamine, 0.6% d-glucose). Then the cells were passed through a 70-μm cell strain before cell count. A total of 60,000 cells/coverslip were plated in 80 μl medium (7.5 × 10^5^ cells/ml) for a 24-well plate. Thirty minutes later, 1 ml of feeding medium (Neurobasal media, 1 × B27 supplement, 1 × penicillin/streptomycin, 1 × l-glutamine) was added into each well.

### Cell Transduction

For cell transduction, at day 2 of cell culture, neurons were transduced with AAV1 vectors of human 4R0N WT tau at MOI 200. The culture medium was replaced ~1/2 volume of feeding media every 3–4 days for cell maintenance until ready to use for experiment on days 14 to 21.

### oTau (S1p) and fTau (P3) Fraction Treatments

S1p and P3 stock solution (20 μg/ml) were diluted in feeding medium for each well in 24-well plates and added into the cells by completely replacing the old medium. Then the cells were fixed by 4% PFA or snap frozen in a time series (3, 24, 48, 96 h) for further analysis.

### Immunofluorescence Labeling of Fixed Primary Culture

**Figure 1:**
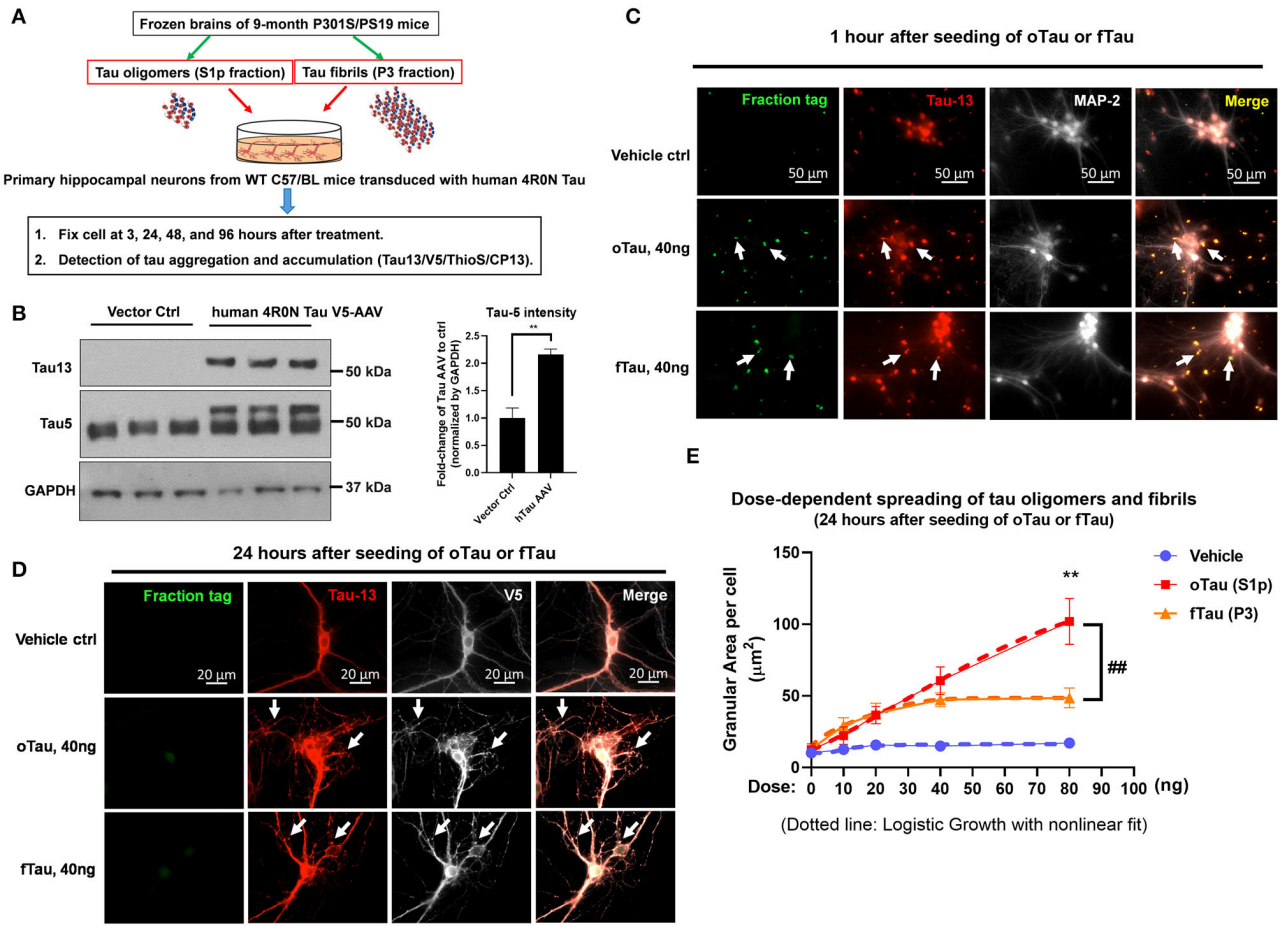
The uptake and self-templating of tau oligomers and fibrils in primary neurons. **(A)** Scheme of experiment design with primary cultures, including the preparation of tau oligomers and fibrils, cell culture plating, transduction, treatment, and harvest in a time course. **(B)** Immunoblot with Tau5 and Tau13 antibody was used to confirm the expression of human tau in transfected C57 neurons. Data are shown as mean ± SEM, data analysis was by two-tailed *t*-test, **p < 0.01. **(C)** Detection for the uptake of DyLight-488 conjugated tau oligomers and fibrils by recipient neurons after 1 h of treatment. The neurons were washed three times with PBS before fixed by 4% PFA and subjected to immunofluorescence labeling. Green is the DyLight-488 conjugated tau oligomers and fibrils. Red is the Tau13 antibody labeled human tau, including the fractions added and the overexpressed human tau in the recipient neurons. Bright is the MAP-2 to label neuronal cells. Scale bar 50 μm. **(D)** The self-templating of tau aggregates in the recipient neurons. Detection for the uptake of DyLight-488 conjugated tau oligomers and fibrils by recipient neurons after 24 h of treatment. At 24 h after the treatment of DyLight-488 conjugated tau oligomers and fibrils, the cells were washed with PBS three times followed by fixation with 4% PFA. Then the digestion of DyLight-488 conjugated tau oligomers and fibrils as well as the self-templating of tau aggregates in the recipient neurons were detected by immunofluorescence labeling. Green is the DyLight-488 conjugated tau oligomers and fibrils. Red is the Tau13 antibody labeled human tau, including the fractions added and the overexpressed human tau in the recipient neurons. Bright is the V5-tagged human tau in the recipients. Scale bar 20 μm. **(E)** The quantification for the dose response of tau oligomer and fibril uptake in the recipient neurons. The dosing was arranged at 0, 10, 20, 40, and 80 ng of tau in each tau oligomer or fibril fractions. The dotted line showed the logistic growth of the accumulated tau oligomers and fibrils with non-linear fit. Data are shown as mean ± SEM, data analysis was by two-way ANOVA, multiple comparison test by Fisher's LSD, **p < 0.01 in comparison with oTau and fTau dosing groups. ##*p* < 0.01 in comparison with the growth line between oTau and fTau dosing groups.

**Figure 2:**
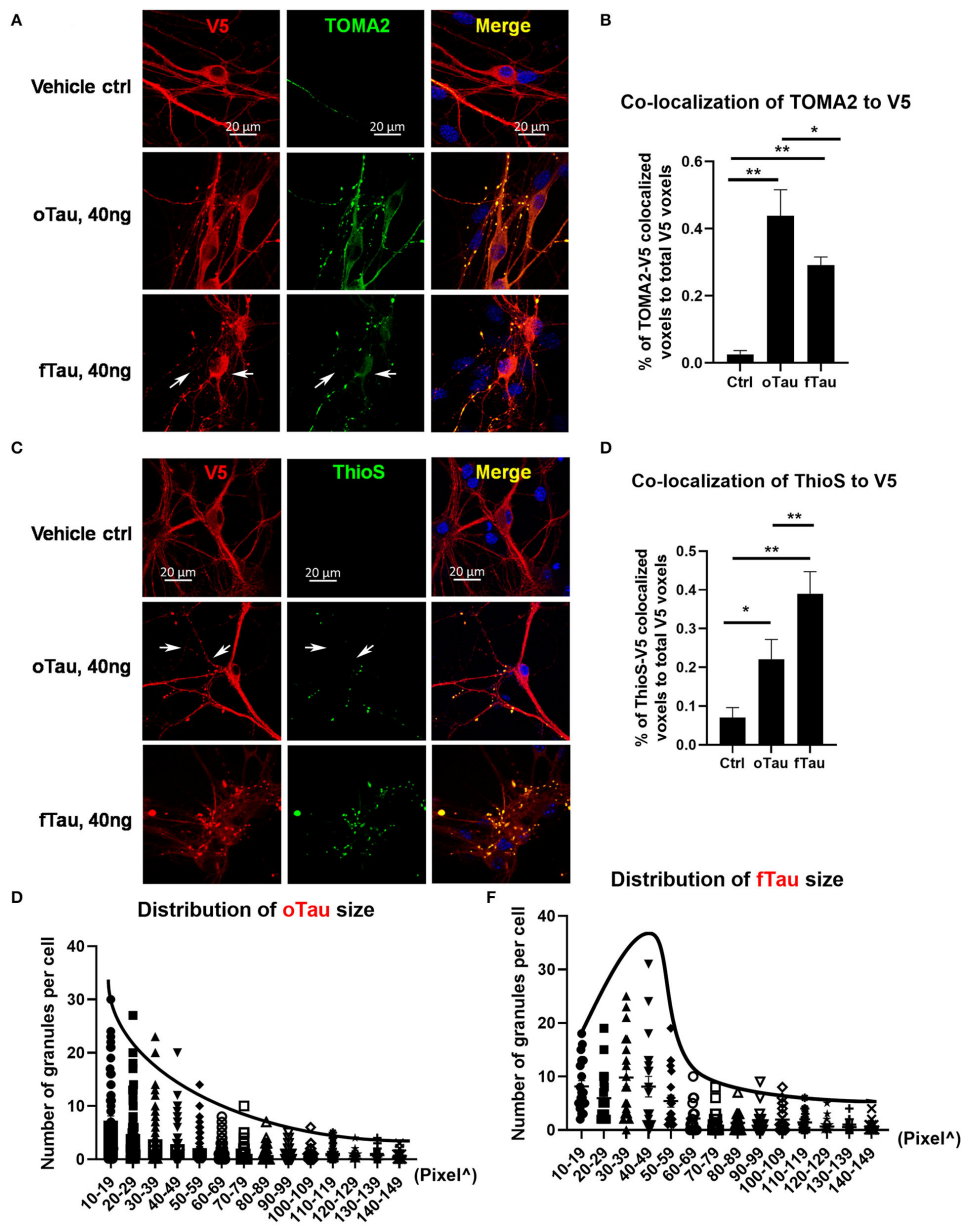
oTau seeds propagate into small tau inclusions while fTau seeds propagate into large tau inclusions. **(A,B)** The hippocampal neurons were treated with oTau (S1p) or fTau (P3) fractions for 24 h. Co-labeling of human tau (V5 tagged, red) in recipient neurons and the oligomeric tau marker TOMA2 (green) showed that seeding of oTau induced accumulation of oligomeric tau, which is significantly higher than in the fTau group. The white arrows point to V5-tagged inclusions present in the fTau group that showed little or no labeling with TOMA2. Data are shown as mean ± SEM, *N* = 20, data analysis was by one-way ANOVA, multiple comparison test by Fisher's LSD, **p < 0.01. Scale bar 20 μm. **(C,D)** The hippocampal neurons were treated with oTau (S1p) or fTau (P3) fractions for 24 h. Co-labeling of human tau (V5 tagged, red) in recipient neurons and the fibril tau marker Thioflavine S (green) showed that seeding of fTau induced accumulation of fibril tau, which is significantly higher than in the oTau group. The white arrows point to V5-tagged inclusions present in the oTau group that showed little or no labeling with Thioflavine S. Data are shown as mean ± SEM, *N* = 20, data analysis was by one-way ANOVA, multiple comparison test by Fisher's LSD, *p < 0.05, **p < 0.01. Scale bar 20 μm. **(E,F)** Analysis of the size distribution of the propagated tau oligomers and fibrils (V5-positive inclusions).

**Figure 3:**
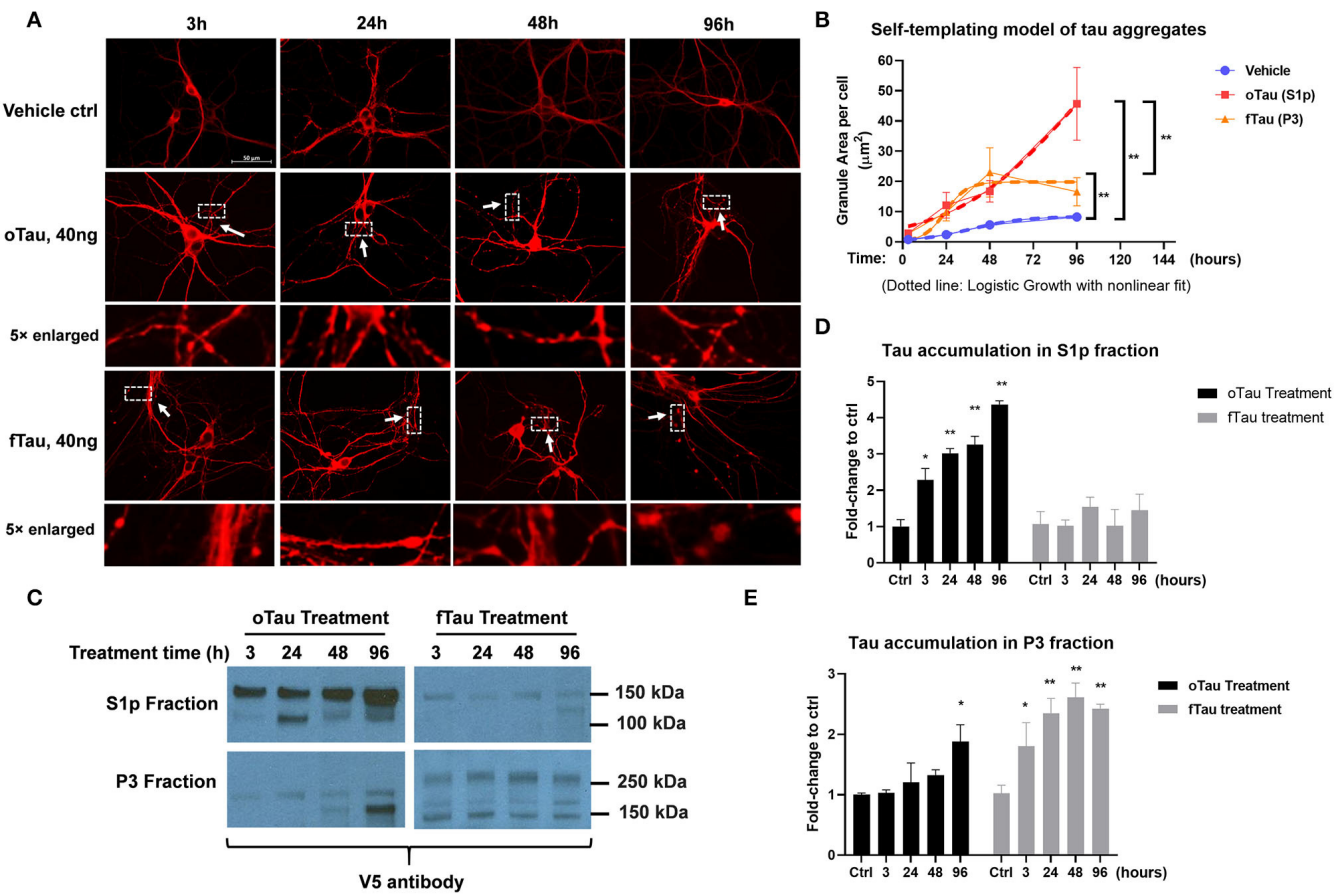
oTau and fTau displayed distinctive propagation cycle in the recipient neurons. **(A)** The representative images of CP13 labeling showed the accumulation of tau inclusions after 3, 24, 48, and 96 h of oTau or fTau seeding. The arrows and boxes highlight regions that are magnified 5-fold immediately below. Scale bar 50 μm. **(B)** Quantification for the accumulation of tau inclusions after seeding of oTau and fTau in a time course of 24, 48, and 96 h after seeding. Dotted lines are the logistic growth with non-linear fit of oTau and fTau in the time course. Data are shown as mean ± SEM, *N* = 30, data analysis was by two-way ANOVA, multiple comparison test by Fisher's LSD, **p < 0.01. **(C)** Immunoblots by V5 antibody after fractionation of S1p and P3 from cells treated with the exogenous seeds oTau or fTau. Cell lysate was collected at 3, 24, 48, and 96 h of oTau or fTau seeding. After extraction of S1p and P3 fractions, 10 μg protein was loaded to the gel for V5-positive tau detection. **(D,E)** Immunoblot quantification of V5-positive tau in S1p and P3 fraction of hippocampal cultures overexpressing human 4R0N WT tau, treated with vehicle control, oTau, or fTau on day 14, and harvested at 3, 24, 48, and 96 h after treatment. Data are shown as mean ± SEM, *N* = 3, data analysis was done by two-way ANOVA, multiple comparison test by Fisher's LSD, *p < 0.05, **p < 0.01 in comparison with vehicle control.

**Figure 4:**
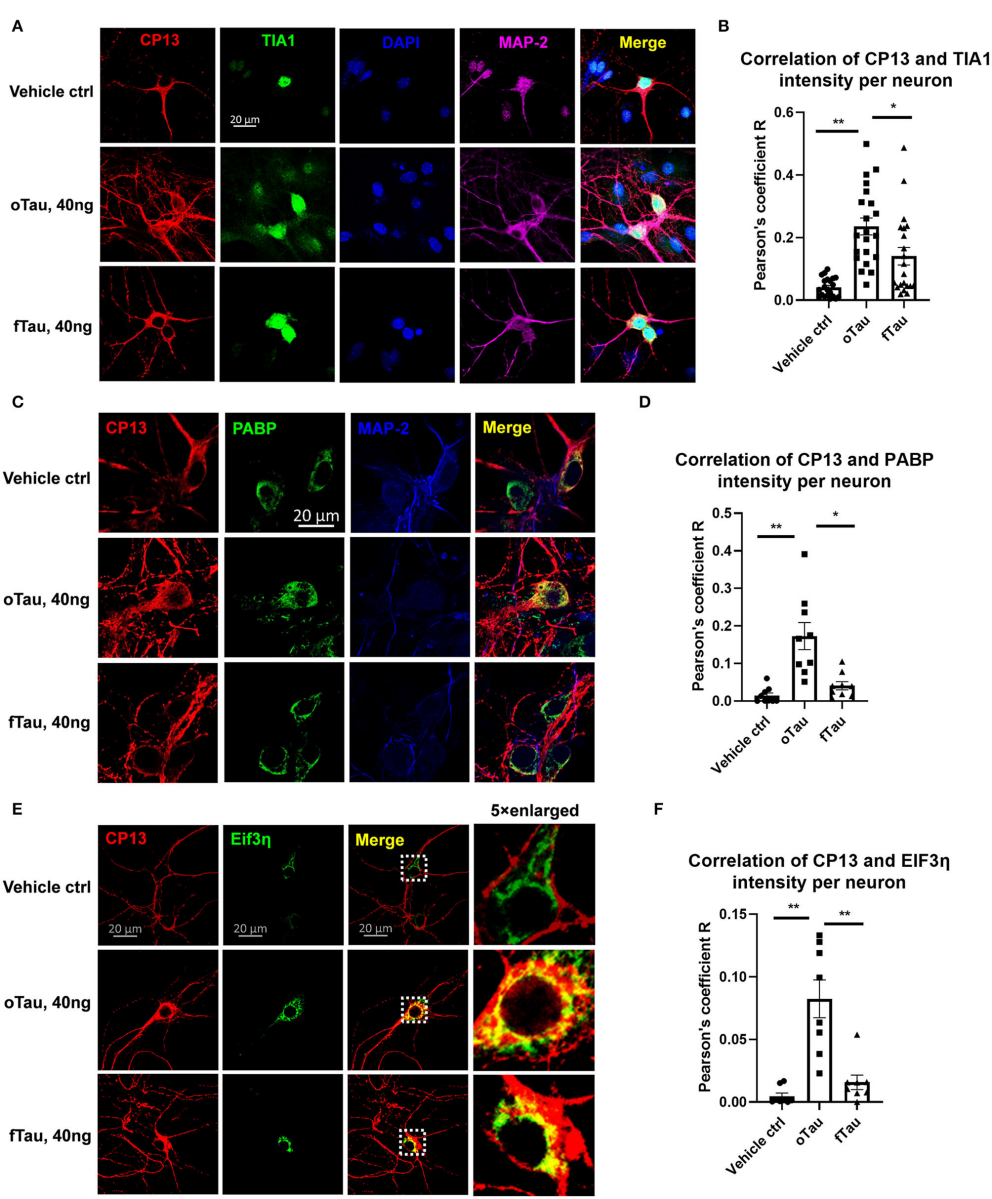
Co-localization of small tau inclusions with stress granules. **(A)** Representative images showing the co-localization of phosphorylated tau inclusions CP13 (red) with TIA1 granules (green) at 3 h after oTau, fTau, or vehicle treatment in hippocampal neurons overexpressing human 4R0N tau. Co-stained markers are MAP-2 (violet) for neuron and DAPI (blue) for nucleus. Scale bar 20 μm. **(B)** Pearson coefficient of correlation between CP13-positive tau with RBP TIA1 is graphed for individual neurons using ImageJ. Data are shown as mean ± SEM, *N* = 20, data analysis was by one-way ANOVA, multiple comparison test by Fisher's LSD, *p < 0.05, **p < 0.01 in comparison with vehicle control. **(C)** Representative images showing the co-localization of phosphorylated tau inclusions CP13 (red) with PABP granules (green) at 3 h after oTau, fTau, or vehicle treatment in hippocampal neurons overexpressing human 4R0N tau. Co-stained marker is MAP-2 (blue) for neuron. Scale bar 20 μm. **(D)** Pearson coefficient of correlation between CP13-positive tau with RBP PABP is graphed for individual neurons using ImageJ. Data are shown as mean ± SEM, *N* = 10, data analysis was done by one-way ANOVA, multiple comparison test by Fisher's LSD, *p < 0.05, **p < 0.01 in comparison with vehicle control. **(E)** Representative images showing the co-localization of phosphorylated tau inclusions CP13 (red) with EIF3η granules (green) at 3 h after oTau, fTau, or vehicle treatment in hippocampal neurons overexpressing human 4R0N tau. The right panel is the 5 × enlarged images to show the detail of co-localization. Scale bar 20 μm. **(F)** Pearson coefficient of correlation between CP13-positive tau with EIF3η is graphed for individual neurons using ImageJ. Data are shown as mean ± SEM, *N* = 10, data analysis was done by one-way ANOVA, multiple comparison test by Fisher's LSD, **p < 0.01 in comparison with vehicle control.

**Figure 5:**
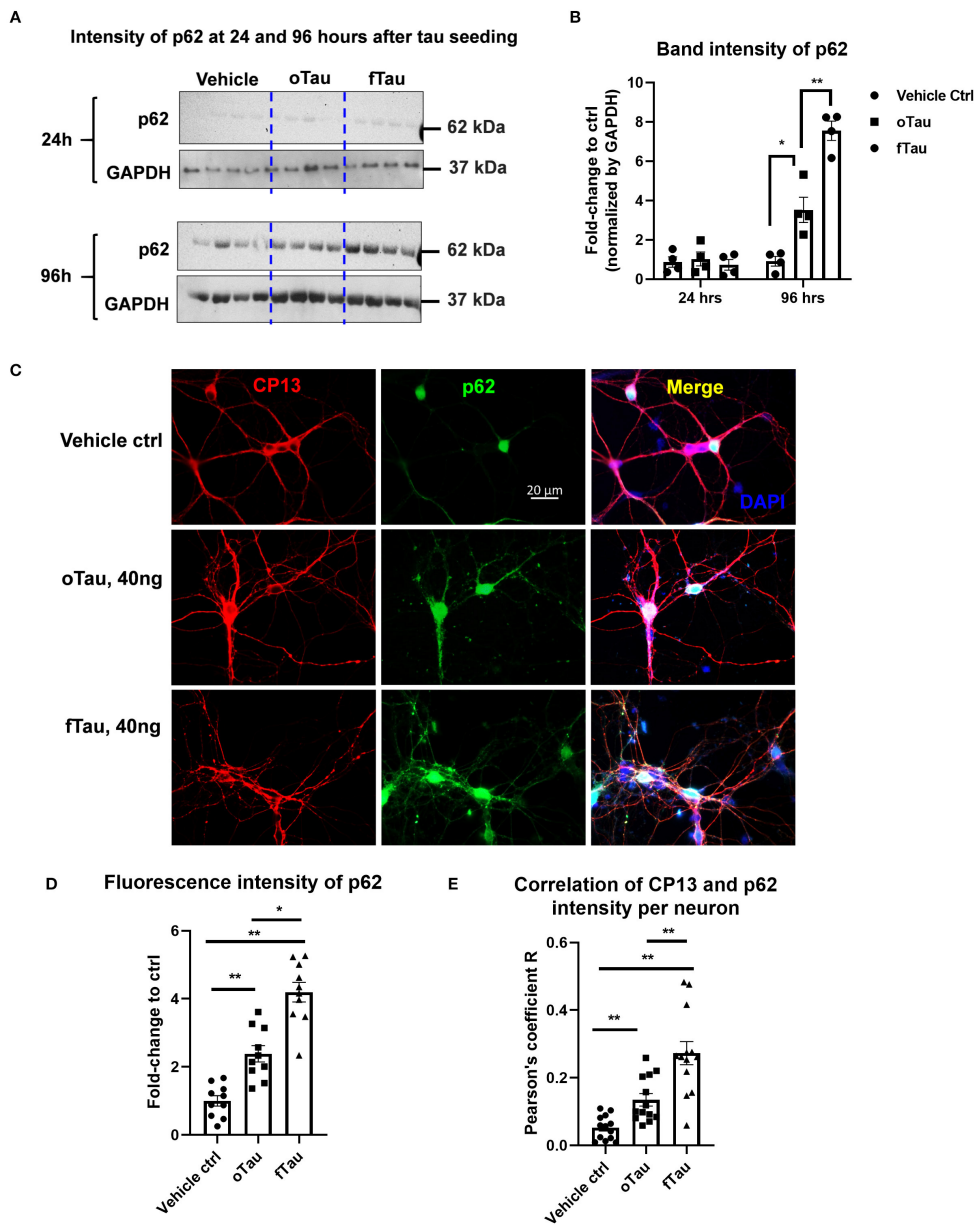
Co-localization of large tau inclusions with p62. **(A)** Immunoblotting detecting p62 expression in neurons after oTau or fTau treatment. Each lane represents an independent biological replicate. The immunoblotting was performed with cell lysate from hippocampal cultures overexpressing human 4R0N WT tau and treated with vehicle, oTau, or fTau. The cells were harvested at 24 and 96 h after treatment. The p62 antibody was used to identify the expression level of p62 protein. **(B)** Quantification of p62 immunoblot in cell lysate of hippocampal cultures overexpressing human 4R0N WT tau, treated with vehicle control, oTau, or fTau on day 14, and harvested at 24 or 96 h after treatment. Data are shown as mean ± SEM, *N* = 4, data analysis was done by two-way ANOVA, multiple comparison test by Fisher's LSD, *p < 0.05, **p < 0.01 in comparison with vehicle control. **(C)** Representative images showing the co-localization of phosphorylated tau inclusions CP13 (red) with p62 granules (green) at 96 h after oTau, fTau, or vehicle treatment in hippocampal neurons overexpressing human 4R0N tau. Co-labeling marker is DAPI (blue) to show the cell nucleus. Scale bar 20 μm. **(D)** Quantification of p62 fluorescence intensity in **(C)**, which is the immunofluorescence labeling of hippocampal cultures overexpressing human 4R0N WT tau, treated with vehicle control, oTau, or fTau on day 14, and harvested at 96 h after treatment. Data are shown as mean ± SEM, *N* = 10, data analysis was done by one-way ANOVA, multiple comparison test by Fisher's LSD, *p < 0.05, **p < 0.01. **(E)** Pearson coefficient of correlation between CP13-positive tau with p62 is graphed for individual neurons using ImageJ. Data are shown as mean ± SEM, *N* = 12, data analysis was done by one-way ANOVA, multiple comparison test by Fisher's LSD, **p < 0.01 in comparison with vehicle control.

Cells on a 24-well coverslip were fixed with 0.5 ml 4% paraformaldehyde (PFA)/phosphate buffered saline (PBS) for 15 min followed by washing three times in PBS. The cells were then permeabilized in 0.5 ml PBS/0.1% Triton X-100 (PBST) for 15–30 min. Blocking was done in 0.5 ml of 5% bovine serum albumin (BSA)−5% donkey serum in PBST for 1 h. The primary antibodies were diluted in 5% BSA/PBST and incubated with the cells overnight at 4 °C. On the 2nd day, the neurons were continued to be incubated in secondary antibodies, which were diluted in 5% BSA/PBST, for 2 h at room temperature (RT). All the secondary antibodies were purchased from Thermo Fisher Scientific made in donkey and used for 1:800 dilution in labeling. After secondary antibody, cells were incubated in DAPI diluted 1:10,000 in PBST (5 mg/ml stock solution) for 5 min after first wash. After washing three times with PBS, coverslips were mounted onto glass microscope slides using 8–10 μl Prolong Gold Antifade mounting media (Thermo Fisher Scientific, cat no. P36930) per coverslip. Slides were naturally dried in fume hood (or stored at 4°C until ready to dry in fume hood). The primary antibodies used in this study for immunocytochemistry are as follows: MAP-2 (chicken, AVES, MAP, 1: 250), MAP-2 (rabbit, Millipore, AB5622, 1:1000), V5 (rabbit, Sigma-Aldrich, V8137, 1:1000), CP-13 (mouse, provided by Peter Davies, 1:300), TIA1 (rabbit, Abcam, ab40693, specifically lot no. GR3202325-1, 1:400), PABP (rabbit, Abcam, ab21060, 1:400), EIF3η (rabbit, Santa Cruz, sc-137214, 1:400), p62 (rabbit, Thermo Fisher Scientific, PA5-27247, 1:400), TOMA2 (mouse, provided by Dr. Rakez Kayed, 1:300), and Tau13 (mouse, provided by Dr. Nicholas M. Kanaan from Michigan State University, 1:1000). Images were captured by a Zeiss Axio Observer microscope in Figures 1, 3, 5. Images were captured by Zeiss LSM700 confocal microscope in Figures 2, 4.

### Immunoblot

For p62 detection in primary culture, cell lysates were collected from frozen cultures with RIPA lysis buffer. Then 40 μg of reducing and non-reducing protein samples was separated by gel electrophoresis and transferred to 0.2-μm nitrocellulose membranes using the Bolt SDS-PAGE system (Life Technologies). Membranes were blocked in 5% non-fat dry milk in PBS supplemented with 0.025% Tween-20 (PBST) for 1 h at RT, followed by incubation overnight at 4°C in primary antibody diluted in 5% BSA/PBST. Primary antibodies used were as follows: Tau13 (mouse, provided by Dr. Nicholas M. Kanaan from Michigan State University, 1:5000); Tau 5 (mouse, provided by Dr. Nicholas M. Kanaan from Michigan State University, 1:500); p62 polyclonal antibody (Invitrogen, cat no. PA5-27247); GAPDH polyclonal antibody (Thermo Fisher Scientific, cat no. PA1987). Membranes were then washed three times with PBST and incubated in horseradish peroxidase–conjugated secondary antibodies (Jackson ImmunoResearch) diluted in 1% BSA/PBST at RT for 1 h. After incubation in secondary antibody, membranes were washed three times in PBST and developed using SuperSignal West Pico Chemiluminescent ECL substrate (Thermo Fisher Scientific, cat no. 34080).

### Image Analysis

To quantify Tau protein aggregates distribution as well as evaluate the colocalization of the Tau protein and the TIA1 stress granules, we developed a customized MATLAB program to process large amounts of immunofluorescence images automatically (Supplementary Figure 1). This algorithm takes the red-fluorescent image that mainly labels the Tau oligomers and fibrils, and has the MATLAB program process the imaging data according to the following procedures: (1) the single-color RGB image is converted to a grayscale image with a bit depth of 8; (2) the noisy background and features are filtered out through the thresholding and the morphological operation; (3) the grayscale image is then converted to a binary image, and the residual filament structures, mostly from the axons, are further removed from this binary image; (4) based on post-processed binary images above, the distribution of Tau protein locations and sizes are calculated, extracted, and exported into a Microsoft Excel file for further statistical analysis. For evaluation of the Tau protein/TIA1 (or PABP, EIF3η) stress granules colocalization, the algorithm processes the red-fluorescent images using the same program to obtain a post-processed binary image. The algorithm also converts the green-fluorescent images into another binary map. Then, the algorithm calculates the overlapping between these two binary images, which determines the percentage of colocalization. There are several predefined parameters applied in the MATLAB programs, which are optimized using iterative testing.

### Statistical Analysis

Statistical analyses were performed using GraphPad Prism version 6.00 for Windows with two-sided α of 0.05. All group data are expressed as mean ± SEM. Column means were compared using one-way ANOVA with treatment as the independent variable. Also, group means were compared using two-way ANOVA with factors on genotype and fraction treatments, respectively. When ANOVA showed a significant difference, pairwise comparisons between group means were examined by Tukey, Dunnett, or uncorrected Fisher's LSD multiple comparison test.

## Results

### Seeding of Exogenous Oligomeric Tau (oTau) and Fibrillar Tau (fTau) Fractions in Recipient Primary Neurons

The experimental is shown in Figure 1A. We extracted the S1p (mainly containing oligomeric tau) and P3 fractions (mainly containing fibrillar tau) from PS19 P301S mice brain aged 9 months. The primary hippocampal neurons were plated on a 24-well plate and transduced with 4R0N human Tau AAV1 on day 2. Overexpression of the 4R0N human tau was utilized to create an abundant pool of endogenous tau that might facilitate propagation. On day 14 of cell culture when the neurons are mature, the cells were treated with S1p (oTau) or P3 (fTau) fractions dosing from 10 to 80 ng, and harvested at the indicated time point for the experiment presented in this article (Figure 1A). Immunoblot with Tau5 and Tau13 antibody confirmed the expression of human tau in transfected C57 neurons (Figure 1B).

To detect the uptake of tau fractions by recipient neurons, the cells were fixed at 1 h after DyLight-488 conjugated tau oligomers or fibril fractions were introduced into the culture medium. The result showed that both oTau and fTau were effectively taken up by neurons (Figure 1C, green); immunocytochemistry with the anti-human tau antibody, Tau13, showed the physical relationship of the exogenous tau to the endogenous human 4R0N tau expressed in these neurons (Figure 1C, red). Next, we repeated the experiment but the primary hippocampal neurons were harvested after 24 h to characterize potential propagation of tau fractions in the recipient neurons. Tau was identified in these cultures by labeling with antibody to human tau (tau13) as well as antibody to a V5 tag, which is also present as part of the chimeric human 4R0N tau expressed in these neurons. Fluorescence from the exogenously applied DyLight-488 conjugated tau oligomers and fibrils had largely faded by 24 h after application (Figure 1D). However, at 24 h, both the S1p and P3-treated groups showed the appearance of abundant V5 positive tau inclusions, corresponding to endogenously produced V5 tagged human tau (Figure 1D). Hippocampal neuron cultures exposed to vehicle exhibited equally strong V5 labeling, but had very smooth labeling without the appearance of inclusions (Figure 1D).

We proceeded to test the dose–response relationship comparing the dose of exogenous tau applied (oTau or fTau) and the resulting level of inclusion formation within the neuron at 24 h. The area per cell covered by the granular tau inclusions at 24 h were quantified after treatment with 0, 10, 20, 40, and 80 ng of oTau or fTau (Figure 1E). For fTau, formation of intracellular tau granules increased up to the 40 ng dose but not beyond (Figure 1E), whereas formation of intracellular tau granules in response to oTau increased linearly at all doses (Figure 1E). Application of non-linear curve fitting by logistic growth analysis showed plateauing for the fTau binding curve, which suggests that the seeding of fTau is limited by the interaction with the rate-limiting binding site or receptor-mediated entry pathway. In contrast, seeding by oTau occurs through a mechanism involving either a high abundance binding site or an entry pathway that does not require binding to a specific receptor (Figure 1E).

### oTau Fractions Seed Small Tau Inclusions While fTau Seed Large Tau Inclusions

We proceeded to compare the distribution of oligomeric, TOMA2-positive tau inclusions with fibrillar, ThioS-positive tau inclusion after exposure to oTau or fTau. The oTau-specific antibody TOMA2 was used to map the distribution of tau oligomers in the recipient neurons at 24 h after tau seeding of oTau or fTau (Figure 2A). V5 antibody was used to label the total tau expression and calculate the fraction of tau inclusions that were TOMA2 positive. These results showed that oTau seeded higher fraction TOMA2-positive tau oligomers than fTau (Figure 2B). Meanwhile, Thioflavine S and V5 co-labeling was performed to detect the fraction of tau inclusions that exhibited fibrillar tau (Figure 2C). These results showed that fTau fraction seeded a higher fraction of fibrillar tau than did oTau (Figure 2D).

We also quantified the size distribution of the tau inclusions in the recipient neurons after 24 h of oTau or fTau seeding. We observed that the number of tau inclusions induced by oTau was highest at the smallest diameters and steadily decreased with size (Figure 2E). In contrast, inclusion induction by fTau peaked at a larger size, with fewer inclusions at the very small or very large size range (Figure 2F).

### oTau and fTau Displayed Distinctive Seeding Cycles in the Recipient Neurons

To determine the replication cycle of tau oligomers and fibrils in the recipient neurons, we seeded the neurons with 40 ng of oTau or fTau, which is a dose that produces similar levels of seeding at 24 h. We then harvested cells over a time course of 3, 24, 48, and 96 h after treatment. The hippocampal neurons were stained with CP13 antibody (pSer202 Tau) to identify the tau inclusions (Figure 3A). By quantifying the granular area in the individual neurons that were treated with oTau and fTau fractions, we found that the accumulation of fTau-induced inclusions plateaued after 48 h, while the accumulation of oTau-induced inclusions continued to grow past 48 h (Figure 3B).

### Small oTau Inclusions Co-Localize to Stress Granules in the Recipient Neurons

Our previous studies found that RNA binding protein TIA1 appeared to stabilize oligomeric tau and reduce conversion into larger fibrillar tau tangles (20). Subsequent studies of tau propagation *in vivo* also suggested that oTau selectively co-localizes with RNA binding proteins (RBPs) linked to SGs, such as TIA1 (19). We preceded to quantitatively compare the association of oTau and fTau with RBPs and SGs in the hippocampal neuron seeding model. We quantified the co-localization of propagated tau with RBPs, including TIA1, PABP, and EIF3η. These RBPs are the key components of SGs which have been found to play important roles in the progression of neurodegenerative diseases (30). Hippocampal neurons overexpressing human 4R0N tau were seeded with oTau or fTau fractions (40 ng/well), and fixed at 3 h after treatment. The cells were co-labeled with antibodies to phospho-tau (CP13) and each RBP. In each case, the result showed stronger co-localization of cytoplasmic RBPs with inclusions induced by oTau than those induced with fTau (Figure 4). The results for TIA1 showed that oTau induced strong TIA1 translocation from the nucleus into neuronal soma and co-localization with oTau inclusions (Figures 4A,B); we also note an overall increase in TIA1 fluorescence intensity in neurons exposed to oTau and fTau. Co-labeling of CP13 with PABP also showed stronger co-localization with oTau-induced inclusions than with inclusions induced by fTau (Figures 4C,D). Labeling with EIF3η also showed the same trend as with PABP; EIF3η showed stronger co-localization with inclusions induced by oTau in neuronal soma than those induced by fTau (Figures 4E,F); we also note an overall increase in EIF3η fluorescence intensity in neurons exposed to oTau and fTau. These results indicate that RNA binding proteins co-localize with the oTau complex.

### The Propagated Large Tau Inclusions Co-Localize to p62 Foci in the Recipient Neurons

Previous studies suggested that fibrillar tau aggregates are recognized by p62/SQSTM1, which are then shunted toward the autolysosomal cascade for degradation (31–33). The results presented earlier suggest that fTau induces large, fibrillar tau inclusions, whereas oTau induces smaller, non-fibrillar inclusions (Figure 3). Because p62 recognizes fibrillar aggregates, we hypothesized that the fibrillar inclusions stimulated by fTau might also be recognized by p62. To test this hypothesis, we harvested the hippocampal neurons at 24 and 96 h after oTau or fTau treatment. Quantification of p62 expression in total cell lysates by immunoblotting showed that there was significantly more p62 expression at 96 h in the neurons seeded with fTau than those seeded with oTau (Figures 5A,B). Next, we examined the degree of co-localization of p62 with tau inclusions at 96 h after seeding with oTau or fTau tau. p62 inclusions were strongly apparent in neurons seeded with either oTau or fTau, and were increased over vehicle-treated neurons (Figures 5C,D). Importantly, p62 was significantly more abundant and showed more co-localization with tau inclusions in neurons seeded with fTau than oTau (Figures 5C,D). These results support the hypothesis that tau inclusions induced by fTau have more fibrillar structures than those induced by oTau, leading to greater recognition by p62 and potentially promoting increased shunting through the autolysosomal cascade.

## Discussion

Protein aggregates show a strong propensity to seed and propagate in cell culture and *in vivo*. Although the morphological changes associated with varying conformers of these aggregates are easily documented, little is known about the biological activities of the varying conformers. In this study, we compared the morphological and biological characteristics of two types of tau aggregates, oTau and fTau. We used tau species generated from 9-month-old P301S tau mice because the oTau generated from these mice is associated with neurodegeneration and previously shown to be toxic (19). We also used primary cultures of hippocampal neurons expressing V5-tagged human tau to increase the rate of seeding and to distinguish between the exogenously applied tau species (that lack a V5 tag) and the endogenously generated tau species that contain a V5 tag. The exogenously applied oTau and fTau were both rapidly transported into the neurons in a dose-dependent manner, but then disappeared within 24 h, consistent with clearance of the exogenous species. In contrast, the resulting inclusions that formed showed labeling by the anti-V5 antibody, demonstrating that these inclusions were predominantly generated from endogenously produced 4R0N tau.

oTau and fTau exhibited striking differences in their uptake and the characteristics of the inclusions subsequently seeded. oTau showed a dose-dependent uptake that plateaued. Such a plateau is typically consistent with receptor binding and saturation of the binding site. Although we did not identify the binding site, a recent study suggests that the lipoprotein receptor LRP1 is an important mediator of tau propagation *in vivo* (34). LRP1 might also mediate uptake of oTau in neuron culture because it is also abundant in primary cultures of hippocampal neurons (35). The uptake of fTau did not exhibit plateauing at the doses used, although we cannot rule out plateauing at higher doses of fTau. The lack of plateauing might reflect uptake through a non-specific mechanism, such as heparin sulfate proteoglycans, as has been reported previously. The differential uptake suggests different mechanisms of uptake between the two species, which has been noted for other aggregates, such as β-amyloid (36). Once taken up, oTau and fTau also elicited different types of inclusions, with the most abundant inclusions induced by oTau being smaller than those induced by fTau. Previous studies of tau seeding consistently note differences in the morphological patterns of seeding associated with each strain of tau conformer (18, 37).

The biological basis for the differing behaviors of tau conformers is typically poorly understood, but in the current case the differential patterns might be linked to clear differences in the resulting processes associated with each type of aggregate. The inclusions induced by fTau were observed to label with ThioS and anti-p62 antibody. Such labeling indicates the presence of amyloids that are recognized by the cell and targeted by p62 to the autolysosomal system for degradation (38–40). The labeling by both ThioS and anti-p62 antibody supports the hypothesis that exogenous fTau seeds endogenous fTau. In contrast to the strong ThioS labeling, the seeded fTau inclusions exhibit relatively modest association with RBPs and SGs, which suggest that they have proportionately modest activity toward the translational stress response and RNA metabolism.

The behavior of oTau seeding contrasts sharply with that of fTau. Inclusions seeded by oTau showed much stronger association with RBPs associated with SGs, including TIA1, PABP, and EIF3η, than with ThioS and p62. This is consistent with previous work showing the association of tau with SGs in cells, in mice, and in human brain (19, 20, 23–28). Overexpressing tau induces SGs, whereas reducing tau appears to inhibit SG formation (21). Studies of mice with reduced TIA1 showed a surprising reduction in oTau but increase in fTau (20). The current study supports this putative linkage between oTau and SG biology, and is consistent with a role for tau in stimulating the translational stress response (30). It seems likely that tau phosphorylation by proline-directed stress kinases, such as GSK-3β, CDK5, and MARK/Par-1, represents an integrated part of this biological pathway. These kinases are all activated by stress, phosphorylation of tau by these kinases stimulates its oligomerization, and phosphorylated oligomeric tau occurs in concert with the translational stress response (9). Thus, tau phosphorylation, tau oligomerization, and the translational stress response are all linked and integrated.

A major question that remains to be definitively answered is causation. Tau oligomerization could result as an unintended byproduct of stress response or could occur as a biological process designed to drive the stress response. It is noteworthy that oligomerization is a normal common biological process that drives many signaling pathways. For instance, oligomerization is a necessary part in the activation of the proteins p53 and p62 (40–43). However, disease-related accumulation of tau oligomers might stimulate the translational stress response as an indirect response to toxicity induced by tau oligomers. Experiments showing that tau reduction protects against β-amyloid toxicity represent a partial step suggesting a biological role for tau in modulating stress, but more work must be done to rigorously determine whether oligomerization of tau presents a bona fide biological function or an unintended toxic byproduct.

## Data Availability Statement

All datasets generated for this study are included in the article/Supplementary Material.

## Ethics Statement

The studies involving animals were reviewed and approved by the Boston University Institutional and Animal Care and Use Committee.

## Author Contributions

LJ and JZ performed the experiments and helped write, conceptualized, and edited the article. J-XC conceptualized and edited the article. BW conceptualized, helped write, and edited the article. All authors contributed to the article and approved the submitted version.

## Conflict of Interest

BW is Co-founder and Chief Scientific Officer for Aquinnah Pharmaceuticals Inc. The remaining authors declare that the research was conducted in the absence of any commercial or financial relationships that could be construed as a potential conflict of interest.

## References

[1] Ballatore C Lee VM Trojanowski JQ. Tau-mediated neurodegeneration in Alzheimer's disease and related disorders. Nat Rev Neurosci. (2007) 8:663–72. 10.1038/nrn2194 17684513

[2] Zempel H Mandelkow E. Lost after translation: missorting of Tau protein and consequences for Alzheimer disease. Trends Neurosci. (2014) 37:721–32. 10.1016/j.tins.2014.08.004 25223701

[3] Christensen KR Beach TG Serrano GE Kanaan NM. Pathogenic tau modifications occur in axons before the somatodendritic compartment in mossy fiber and Schaffer collateral pathways. Acta Neuropathol Commun. (2019) 7:29. 10.1186/s40478-019-0675-9 30819250 PMC6394076

[4] Friedhoff P Schneider A Mandelkow EM Mandelkow E. Rapid assembly of Alzheimer-like paired helical filaments from microtubule-associated protein tau monitored by fluorescence in solution. Biochemistry. (1998) 37:10223–30. 10.1021/bi980537d 9665729

[5] Kampers T Friedhoff P Biernat J Mandelkow EM Mandelkow E. RNA stimulates aggregation of microtubule-associated protein tau into Alzheimer-like paired helical filaments. FEBS Lett. (1996) 399:344–9. 10.1016/S0014-5793(96)01386-5 8985176

[6] Wilson DM Binder LI. Free fatty acids stimulate the polymerization of tau and amyloid beta peptides. *In vitro* evidence for a common effector of pathogenesis in Alzheimer's disease. Am J Pathol. (1997) 150:2181–95. 9176408 PMC1858305

[7] Dobson CM. Protein folding and misfolding. Nature. (2003) 426:884–90. 10.1038/nature02261 14685248

[8] Selkoe DJ Hardy J. The amyloid hypothesis of Alzheimer's disease at 25 years. EMBO Mol Med. (2016) 8:595–608. 10.15252/emmm.201606210 27025652 PMC4888851

[9] Stoothoff WH Johnson GV. Tau phosphorylation: physiological and pathological consequences. Biochim Biophys Acta. (2005) 1739:280–97. 10.1016/j.bbadis.2004.06.017 15615646

[10] Fitzpatrick AWP Falcon B He S Murzin AG Murshudov G Garringer HJ. Cryo-EM structures of tau filaments from Alzheimer's disease. Nature. (2017) 547:185–90. 10.1038/nature23002 28678775 PMC5552202

[11] Falcon B Zivanov J Zhang W Murzin AG Garringer HJ Vidal R. Novel tau filament fold in chronic traumatic encephalopathy encloses hydrophobic molecules. Nature. (2019) 568:420–423. 10.1038/s41586-019-1026-5 30894745 PMC6472968

[12] Falcon B Zhang W Murzin AG Murshudov G Garringer HJ Vidal R. Structures of filaments from Pick's disease reveal a novel tau protein fold. Nature. (2018) 561:137–40. 10.1038/s41586-018-0454-y 30158706 PMC6204212

[13] Kellogg EH Hejab NMA Poepsel S Downing KH DiMaio F Nogales E. Near-atomic model of microtubule-tau interactions. Science. (2018) 360:1242–6. 10.1126/science.aat1780 29748322 PMC6225777

[14] Arakhamia T Lee CE Carlomagno Y Duong DM Kundinger SR Wang K. Posttranslational modifications mediate the structural diversity of tauopathy strains. Cell. (2020) 180:633–44.e12. 10.1016/j.cell.2020.01.027 32032505 PMC7491959

[15] Santacruz K Lewis J Spires T Paulson J Kotilinek L Ingelsson M. Tau suppression in a neurodegenerative mouse model improves memory function. Science. (2005) 309:476–81. 10.1126/science.1113694 16020737 PMC1574647

[16] Crimins JL Rocher AB Luebke JI. Electrophysiological changes precede morphological changes to frontal cortical pyramidal neurons in the rTg4510 mouse model of progressive tauopathy. Acta Neuropathol. (2012) 124:777–95. 10.1007/s00401-012-1038-9 22976049 PMC3509230

[17] Rocher AB Crimins JL Amatrudo JM Kinson MS Todd-Brown MA Lewis J. Structural and functional changes in tau mutant mice neurons are not linked to the presence of NFTs. Exp Neurol. (2010) 223:385–93. 10.1016/j.expneurol.2009.07.029 19665462 PMC2864360

[18] Sanders DW Kaufman SK DeVos SL Sharma AM Mirbaha H Li A. Distinct tau prion strains propagate in cells and mice and define different tauopathies. Neuron. (2014) 82:1271–88. 10.1016/j.neuron.2014.04.047 24857020 PMC4171396

[19] Jiang L Ash PEA Maziuk BF Ballance HI Boudeau S Abdullatif AA. TIA1 regulates the generation and response to toxic tau oligomers. Acta Neuropathol. (2019) 137:259–77. 10.1007/s00401-018-1937-5 30465259 PMC6377165

[20] Apicco DJ Ash PEA Maziuk B LeBlang C Medalla M Al Abdullatif A. Reducing the RNA binding protein TIA1 protects against tau-mediated neurodegeneration *in vivo*. Nat Neurosci. (2018) 21:72–80. 10.1038/s41593-017-0022-z 29273772 PMC5745051

[21] Vanderweyde T Apicco DJ Youmans-Kidder K Ash PE Cook C Lummertz da Rocha E. Interaction of tau with the RNA-binding protein TIA1 regulates tau pathophysiology and toxicity. Cell Rep. (2016) 15:1–12. 10.1016/j.celrep.2016.04.045 27160897 PMC5325702

[22] Vanderweyde T Yu H Varnum M Liu-Yesucevitz L Citro A Ikezu T. Contrasting pathology of stress granule proteins TIA-1 and G3BP in tauopathies. J Neurosci. (2012) 32:8270–83. 10.1523/JNEUROSCI.1592-12.2012 22699908 PMC3402380

[23] Koren S Galvis-Escobar S Abisambra JF. Tau-mediated dysregulation of RNA: evidence for a common molecular mechanism of toxicity in frontotemporal dementia and other tauopathies. Neurobiol Dis. (2020) 141:104939. 10.1016/j.nbd.2020.104939 32413399 PMC7397812

[24] Meier S Bell M Lyons DN Rodriguez-Rivera J Ingram A Fontaine SN. Pathological Tau promotes neuronal damage by impairing ribosomal function and decreasing protein synthesis. J Neurosci. (2016) 36:1001–7. 10.1523/JNEUROSCI.3029-15.2016 26791227 PMC4719006

[25] Silva JM Rodrigues S Sampaio-Marques B Gomes P Neves-Carvalho A Dioli C. Dysregulation of autophagy and stress granule-related proteins in stress-driven Tau pathology. Cell Death Differ. (2019) 26:1411–27. 10.1038/s41418-018-0217-1 30442948 PMC6748085

[26] Sengupta U Montalbano M McAllen S Minuesa G Kharas M Kayed R. Formation of toxic oligomeric assemblies of rna-binding protein: musashi in alzheimer's disease. Acta Neuropathol Commun. (2018) 6:113. 10.1186/s40478-018-0615-0 30367664 PMC6203984

[27] Younas N Zafar S Shafiq M Noor A Siegert A Arora AS. SFPQ and tau: critical factors contributing to rapid progression of Alzheimer's disease. Acta Neuropathol. (2020) 140:317–39. 10.1007/s00401-020-02178-y 32577828 PMC7423812

[28] Maziuk BF Apicco DJ Cruz AL Jiang L Ash PEA da Rocha EL. RNA binding proteins co-localize with small tau inclusions in tauopathy. Acta Neuropathol Commun. (2018) 6:71. 10.1186/s40478-018-0574-5 30068389 PMC6069705

[29] Maina MB Bailey LJ Wagih S Biasetti L Pollack SJ Quinn JP. The involvement of tau in nucleolar transcription and the stress response. Acta Neuropathol Commun. (2018) 6:70. 10.1186/s40478-018-0565-6 30064522 PMC6066928

[30] Wolozin B Ivanov P. Stress granules and neurodegeneration. Nat Rev Neurosci. (2019) 20:649–66. 10.1038/s41583-019-0222-5 31582840 PMC6986315

[31] Moreau K Fleming A Imarisio S Lopez Ramirez A Mercer JL Jimenez-Sanchez M. PICALM modulates autophagy activity and tau accumulation. Nat Commun. (2014) 5:4998. 10.1038/ncomms5998 25241929 PMC4199285

[32] Zhang Y Chen X Zhao Y Ponnusamy M Liu Y. The role of ubiquitin proteasomal system and autophagy-lysosome pathway in Alzheimer's disease. Rev Neurosci. (2017) 28:861–8. 10.1515/revneuro-2017-0013 28704199

[33] Fu H Possenti A Freer R Nakano Y Hernandez Villegas NC Tang M. A tau homeostasis signature is linked with the cellular and regional vulnerability of excitatory neurons to tau pathology. Nat Neurosci. (2019) 22:47–56. 10.1038/s41593-018-0298-7 30559469 PMC6330709

[34] Rauch JN Luna G Guzman E Audouard M Challis C Sibih YE. LRP1 is a master regulator of tau uptake and spread. Nature. (2020) 580:381–5. 10.1038/s41586-020-2156-5 32296178 PMC7687380

[35] Sen A Alkon DL Nelson TJ. Apolipoprotein E3 (ApoE3) but not ApoE4 protects against synaptic loss through increased expression of protein kinase C epsilon. J Biol Chem. (2012) 287:15947–58. 10.1074/jbc.M111.312710 22427674 PMC3346146

[36] Yu Y Ye RD. Microglial Abeta receptors in Alzheimer's disease. Cell Mol Neurobiol. (2015) 35:71–83. 10.1007/s10571-014-0101-6 25149075 PMC11486233

[37] Jackson SJ Kerridge C Cooper J Cavallini A Falcon B Cella CV. Short fibrils constitute the major species of seed-competent tau in the brains of mice transgenic for human P301S Tau. J Neurosci. (2016) 36:762–72. 10.1523/JNEUROSCI.3542-15.2016 26791207 PMC4719013

[38] Bjorkoy G Lamark T Brech A Outzen H Perander M Overvatn A. p62/SQSTM1 forms protein aggregates degraded by autophagy and has a protective effect on huntingtin-induced cell death. J Cell Biol. (2005) 171:603–14. 10.1083/jcb.200507002 16286508 PMC2171557

[39] Pankiv S Clausen TH Lamark T Brech A Bruun JA Outzen H. p62/SQSTM1 binds directly to Atg8/LC3 to facilitate degradation of ubiquitinated protein aggregates by autophagy. J Biol Chem. (2007) 282:24131–45. 10.1074/jbc.M702824200 17580304

[40] Wurzer B Zaffagnini G Fracchiolla D Turco E Abert C Romanov J. Oligomerization of p62 allows for selection of ubiquitinated cargo and isolation membrane during selective autophagy. Elife. (2015) 4:e08941. 10.7554/eLife.08941.024 26413874 PMC4684078

[41] Fischer NW Prodeus A Malkin D Gariepy J. p53 oligomerization status modulates cell fate decisions between growth, arrest and apoptosis. Cell Cycle. (2016) 15:3210–19. 10.1080/15384101.2016.1241917 27754743 PMC5176156

[42] Sun XJ Wang Z Wang L Jiang Y Kost N Soong TD. A stable transcription factor complex nucleated by oligomeric AML1-ETO controls leukaemogenesis. Nature. (2013) 500:93–7. 10.1038/nature12287 23812588 PMC3732535

[43] Nguyen TV Yao S Wang Y Rolfe A Selvaraj A Darman R. The R882H DNMT3A hot spot mutation stabilizes the formation of large DNMT3A oligomers with low DNA methyltransferase activity. J Biol Chem. (2019) 294:16966–77. 10.1074/jbc.RA119.010126 31582562 PMC6851320

