# Supplementary material for: Tau Oligomers and Fibrils Exhibit Differential Patterns of Seeding and Association With RNA Binding Proteins
Source: Front Neurol. 2020 Sep 30;11:579434. doi: 10.3389/fneur.2020.579434 (PMC7554625; doi:10.3389/fneur.2020.579434)
Supplement: Supplementary file 1 [file Data_Sheet_1.pdf]

Supplemental Figures

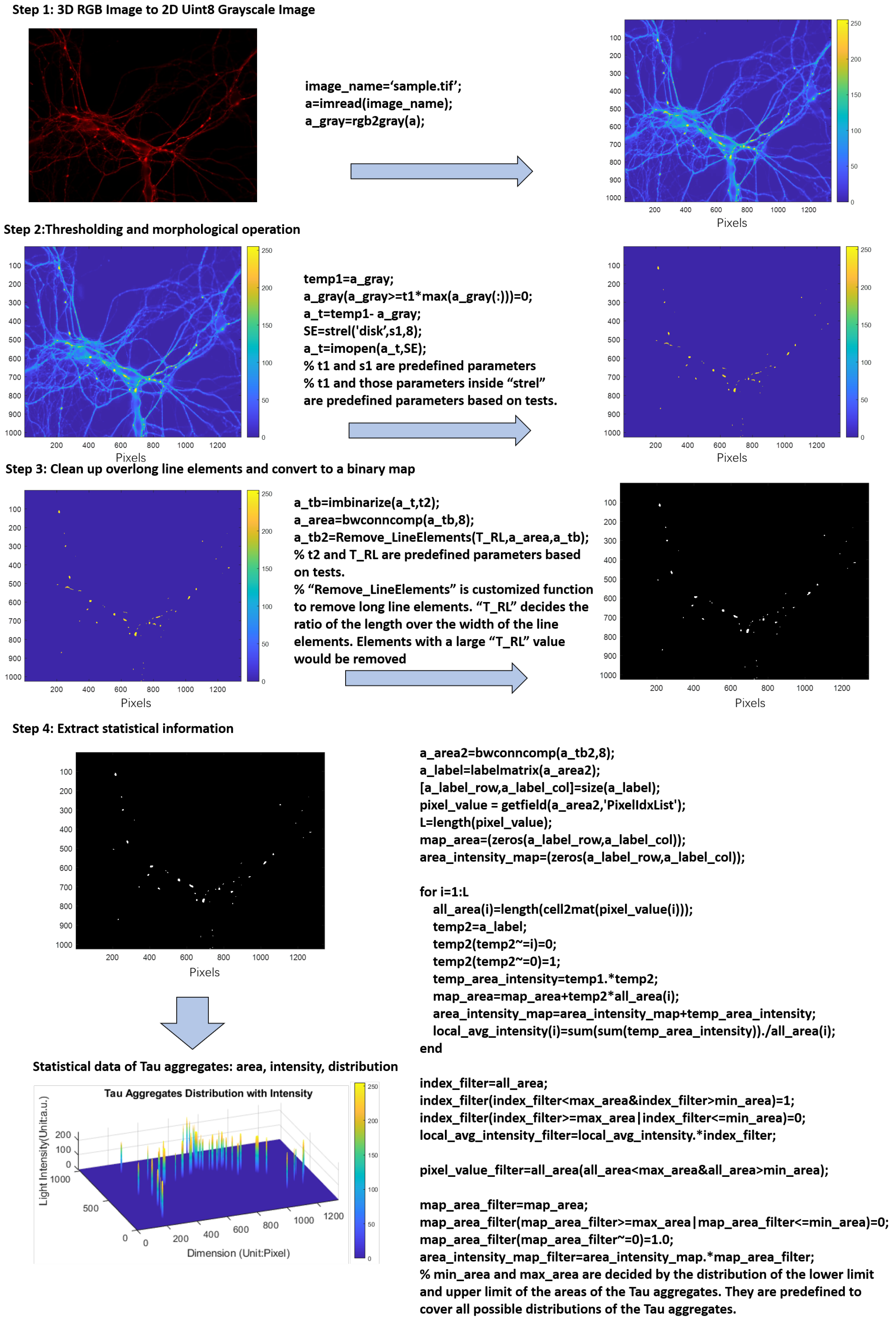

Supplemental Figure S1. Workflow for Tau aggregate extractions from an immune-fluorescence image using MATLAB.

In Fig. S1, we show the main steps used to extract Tau aggregates from a sample immune-

fluorescence image using the MATLAB coding. The key MATLAB commands are listed in each step. The contrast and image sharpness affect the final output results. Accurate analysis requires high-quality experimental imaging data with good contrast and high resolution. Those images that are out of focus and demonstrate a very low contrast would degrade the reliability of the data processing. The predefined parameters are decided by testing on a subset of the experimental data. To automatically process a large amount of data, the key commands shown in Fig. S1 can be referenced in a MATLAB loop repeatedly.

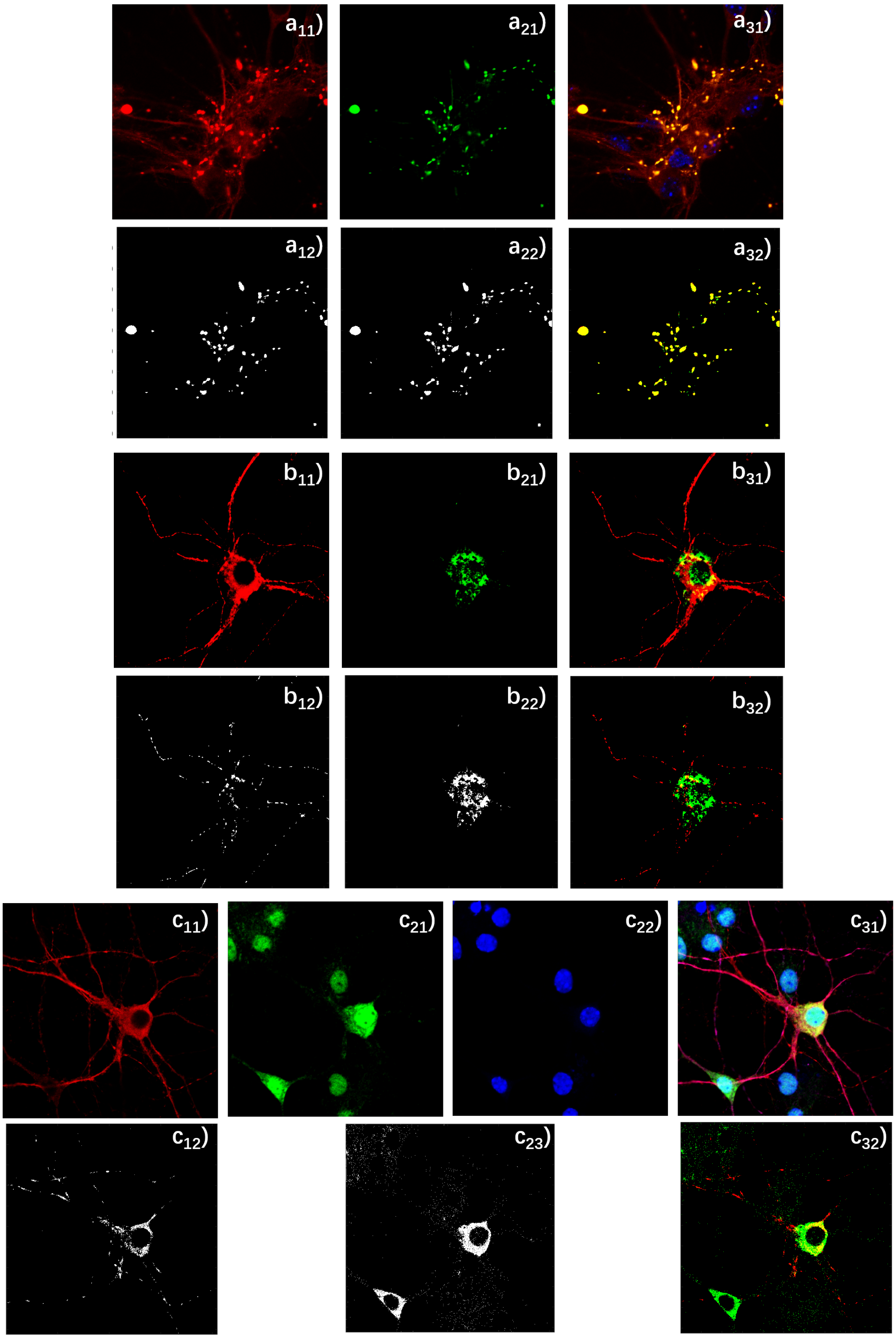

Supplemental Figure S2. Co-localization analysis by MATLAB. a_{11})-a_{31}) are the fluorescence images obtained through V5 and ThioS co-staining; b_{11})-b_{31}) are the fluorescence images obtained through CP13 and EIF3η; c_{11}), c_{21}), c_{22}), and c_{31}) are the fluorescence images obtained through CP13 and TIA1. a_{12}), b_{12}) and c_{12}) are the binary images obtained through the workflow shown in Fig. S1. using the corresponding red images. a_{22}), b_{22}) are the binary images directly transformed from the corresponding green images without additional processing. c_{23}) is the binary image obtained by subtracted c_{22}) from c_{21}) using their binary transformations. a_{32}), b_{32}) and c_{32}) shows the overlapping of the processed data. The yellow color indicates the overlapping area that can be compared with that of the experimental data shown in a_{31}), b_{31}), and c_{31}).

The work flow for Supplemental Figure S2: Representative figures showing the co-localization results produced with our method. The red images in Fig. S2 are processed based on the workflow shown in Fig. S1. The green images in Fig. S2 a_{21} and b_{21} are directly transformed into binary masks (a_{22} and b_{22}) by setting the non-zero values to "1" after removing background noise through thresholding. The red and green images (c_{11} and c_{21}) are transformed into binary masks (c_{12} is the binary mask from red image, while the binary mask from c_{21} is not shown here) as described for the figure series "a" and "b". Then, the blue images in c_{22} used to identify nuclei are shown, but the blue area is ultimately extracted from the corresponding areas of the binary mask obtained from c_{21} to create c_{23}. Finally, the binary mask obtained from the red image (a_{12},b_{12}, and c_{12}) and the binary mask (a_{22}, b_{22}, and c_{23}) are loaded into the red channel and green channel of a three-dimensional matrix, respectively. Then, the RGB image is displayed through this matrix so that the overlapping areas display yellow color, as shown in a_{32}, b_{32}, and c_{32}.

Supplemental Figure S3

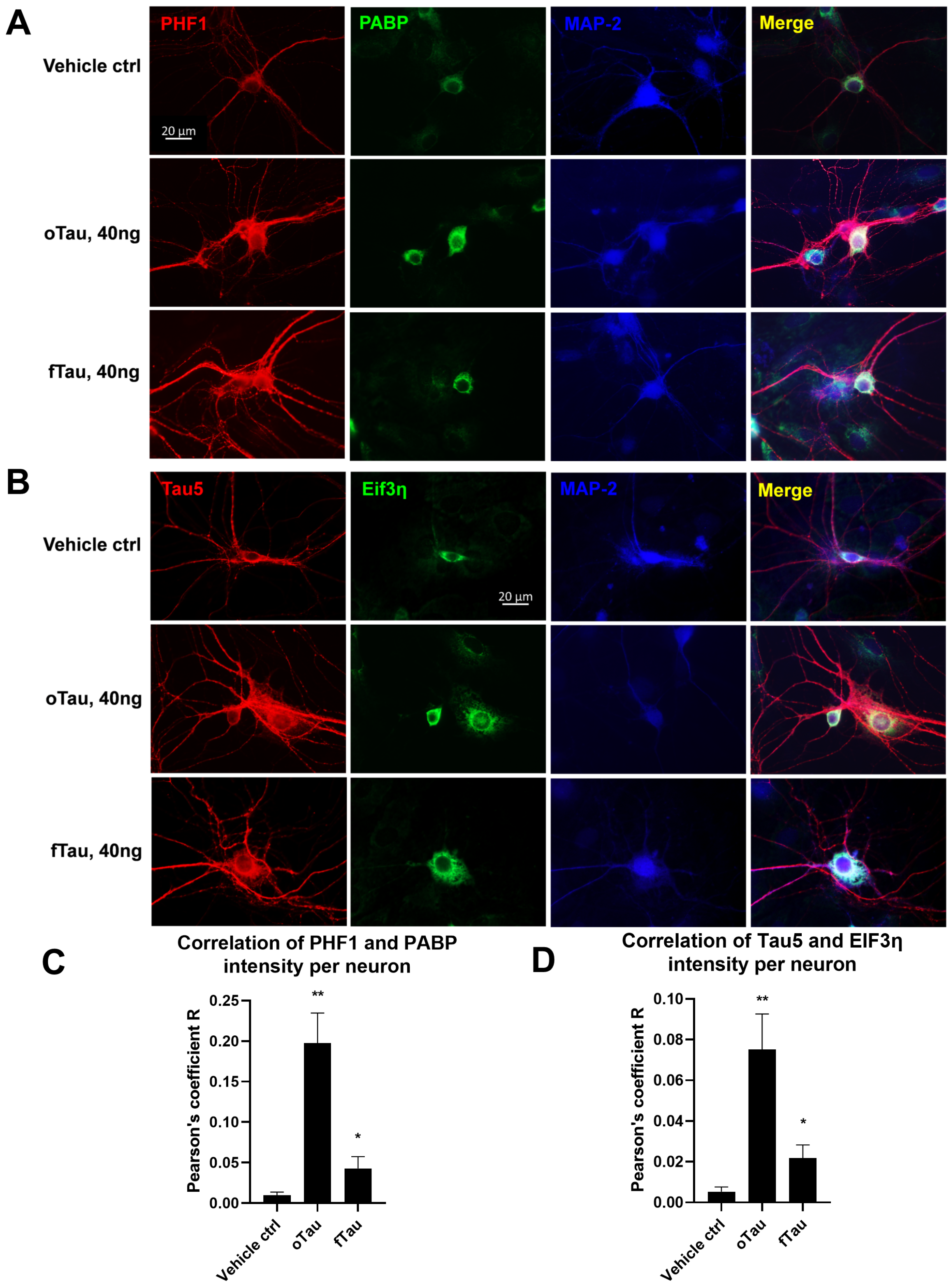

Supplemental Figure S3. co-localization of small tau inclusions with stress granules.
A. Representative images showing the co-localization of phosphorylated tau inclusions PHF1

(red) with PABP granules (green) at 3 hours after oTau, fTau or vehicle treatment in hippocampal neurons over-expressing human 4R0N tau. MAP-2 (blue) is used as a marker to co-label neuron. Scale bar 20μm.

B. Pearson coefficient of correlation between PHF1 positive tau with RBP PABP is graphed for individual neurons using ImageJ. Data were shown as mean ± SEM, N=8, data analysis was done by one-way ANOVA, multiple comparison test by Fisher's LSD, *p<0.05, **p<0.01 in comparison to vehicle control.

C. Representative images showing the co-localization of total tau inclusions Tau5 (red) with EIF3η granules (green) at 3 hours after oTau, fTau or vehicle treatment in hippocampal neurons over-expressing human 4R0N tau. Scale bar 20μm.

D. Pearson coefficient of correlation between Tau5 positive tau with EIF3η is graphed for individual neurons using ImageJ. Data were shown as mean ± SEM, N=8, data analysis was done by one-way ANOVA, multiple comparison test by Fisher's LSD, *p<0.05, **p<0.01 in comparison to vehicle control.
